# Biocontrol of early blight disease of eggplant using endophytic *Aspergillus terreus*: improving plant immunological, physiological and antifungal activities

**DOI:** 10.1186/s40529-022-00357-6

**Published:** 2022-08-28

**Authors:** Mohamed S. Attia, Amr H. Hashem, Ali A. Badawy, Amer M. Abdelaziz

**Affiliations:** 1 Botany and Microbiology Department, Faculty of Science, Al-Azhar University, Nasr City, Cairo, 11884 Egypt; 2 Botany and Microbiology Department, Faculty of Science, Al-Azhar University, Nasr City, Cairo, 11884 Egypt

**Keywords:** Eggplant growth, *Alternaria solani*, Early blight, Osmolytes, Antioxidant enzymes, Isozymes, Endophytes

## Abstract

**Background:**

The eggplant suffers from many biotic stresses that cause severe damage to crop production. One of the most destructive eggplant pathogens is *Alternaria solani*, which causes early blight disease. A pot experiment was conducted to evaluate the role of fungal endophytes in protecting eggplant against early blight as well as in improving its growth performance.

**Results:**

Endophytic *Aspergillus terreus* was isolated from *Ocimum basilicum* leaves and identified morphologically and genetically. In vitro, crude extract of endophytic *A. terreus* exhibited promising antifungal activity against *A. solani* where minimum inhibitory concentration (MIC) was 1.25 mg/ml. Severity of the disease and rate of protection from the disease were recorded. Vegetative growth indices, physiological resistance signs (photosynthetic pigments, carbohydrates, proteins, phenols, proline, malondialdehyde (MDA), antioxidant enzymes), and isozymes were estimated. *Alternaria solani* caused a highly disease severity (87.5%) and a noticeable decreasing in growth characteristics and photosynthetic pigments except for carotenoids. Also, infection with *A. solani* caused significant decreases in the contents of carbohydrate and protein by 29.94% and 10.52%, respectively. Infection with *A. solani* caused enhancement in phenolics (77.21%), free proline (30.56%), malondialdehyde (30.26%), superoxide dismutase (SOD) (125.47%), catalase (CAT) (125.93%), peroxidase (POD) (25.07%) and polyphenol oxidase (PPO) (125.37%) compared to healthy plants. In contrast, the use of *A. terreus* on infected plants succeeded in recovering eggplants from the disease, as the disease severity was recorded (caused protection by 66.67%). Application of *A. terreus* either on healthy or infected eggplants showed several responses in number and density of peroxidase (POD) and polyphenol oxidase (PPO) isozymes.

**Conclusion:**

It is necessary for us to address the remarkable improvement in the photosynthetic pigments, protein, carbohydrates, and enzymatic activity compared to infected control, which opens the way for more studies on the use of biocides as safe alternatives against fungal diseases.

## Introduction

Eggplant (*Solanum melongena*) is one of the most important crops all over the world. It is distinguished by its fleshy fruits, and it is ranked second after potatoes. Where the total global production of it is more than 32 million tons (Choudhary and Gaur 2009; Alhaithloul et al. 2019). Eggplant is distinguished by containing large proportions of protein (1.4 g), carbohydrates (4 g) and calcium (18 mg), in addition to many vitamins; the most important of which are A, B and C vitamins (Butnariu and Butu 2015). Egypt is the third country worldwide in eggplant production (Abdelaziz et al. 2022c; Mat Sulaiman et al. 2020). Eggplant crop faces many pests and diseases that cause severe damage to production. The losses more than 50% in eggplant production resulting from fungal infection (Tsitsigiannis et al. 2008). The world is currently suffering from multiple crises, the most important of which are climate changes, which have helped many pathogens spread and affect food productivity. Also, the frequent use of chemical pesticides has revealed many problems in plant, human, and animal health. It is now necessary for researchers to try to reach biological methods as safe alternatives to the use of chemical pesticides (Abdelaziz et al. 2022a; Attia et al. 2016). Previous studies have proven that the use of plant growth-promoting microorganisms has a significant anti-microbial effect in addition to stimulating the plant’s physiological immunity, which is beneficial to increasing productivity (Attia et al. 2022a, 2022b; Abd Alhakim et al. 2022).

Fungal diseases are among the most dangerous biological stresses that cause severe damage to agricultural crops in Egypt (Aldinary et al. 2021; Farrag et al. 2017; Abdelaziz et al. 2022c, 2021; Attia et al. 2016). Fungal phytopathogens cause harmful effects on crops quality and quantity that may be recovered by induction of plant biochemical defense through nonpathogenic fungi (Abd Alhakim et al. 2022). One of the most famous pathogens of fungal diseases, *Alternaria*, causes a negative impact on crops, especially vegetable crops (Mancini and Romanazzi 2014). *Alternaria* is one of the most common pathogenic fungi that causes early blight and leads to a sharp decline in yield. The infection begins on the lower leaves of the plant first and then extends upwards, where small circular spots with demarcated edges of dark brown or black with diameters ranging between 2–4 mm appear on the leaves and inside of which there are overlapping circular rings. The spots are surrounded by faint halos of plant tissue the spot expands to cover the surface of the leaf and when fused together the leaves dry and fall off (Gupta et al. 2017; Logrieco et al. 2009). The spots may increase during the season and under conditions suitable for the disease, where the spots on the leaves unite, dry, and die. The plant may become barren, and the fruits are exposed to sunstroke. The stem spots on the seedlings are small and slightly sunken; they grow up and turn into circular or elongated spots with concentrated rings in them. If seedlings infected with stem spots are planted, the spots grow especially in the crown or neck area and encircle the leg (Tsedaley 2014).

Plants can induce disease resistance to a group of pathogenic and non-pathogenic microorganisms to those plants or to varieties of them (Moisan et al. 2019). Enhancement of plant resistance may be induced by biotic and abiotic elicitors (Farrag et al. 2017; Omer et al. 2022; Elbasuney et al. 2022). The soil properties depending mainly on biological profile that can highly affect on plant growth and tolerance (Khalil et al. 2015; Eid et al. 2021). It is worth noting that endophytic microorganisms are one of the most important biological means for stimulating and encouraging plant immunity against biotic and a biotic stress (Abdelaziz et al. 2022b; Aldinary et al. 2021; Salem and Husen 2023; Alnusairi et al. 2022; Eid et al. 2019). Endophytic Aspergilli are promising reservoirs for bioactive compounds (Sharaf et al. 2022). Endophytic *A. terreus* has an interesting antifungal activity against fungal pathogens due to its extract contains 16 vital bioactive compounds (Hashem et al. 2022). Application of endophytic *Aspergillus* on infected plants resulted in a significant increase in the content of photosynthetic pigments, total proteins, total carbohydrates, and total phenols in comparison with the infected untreated control plants (Aldinary et al. 2021). Endophytes are microorganisms that produce improved, growth-stimulating, and anti-microbial compounds that grow naturally within plants (Iqbal and Ansari 2020). Application of *Aspergillus* as an endophytic fungus has been documented to enhance chlorophyll contents, morphological growth attributes in different stressed crops; via regulating the osmolytes and antioxidant enzymes activities (Asaf et al. 2018; Badawy et al. 2021b). Hence, the use of endophytes in stimulating the physiological and synthetic immunity of plants was one of the most important biological factors in resistance and increasing crop yield (Adeleke and Babalola 2021; Sturz et al. 2000).

Pesticides are the anti-fungal capabilities of the endophyte *A. terreus* chemical or natural substances or a mixture of them that are used to limit the spread of pathogens that attack the plant and to control those pathogens through their action on the nervous system, stomach and respiratory system of insects or on the process of multiplication and division of other microbes (Oguh et al. 2019). The fungicide Difenoconazole is used for field treatment and protection of plants from phytopathogenic fungi (Elansky et al. 2016). This study aims to evaluate and compare, and the chemical fungicide Daify Core (Difenoconazole 25%) on infected eggplant with *A. solani*, which increases plant resistance as well as the growth and physiological characteristics to face this biotic stress. So, endophyte *A. terreus* consider therapeutic nutrients that have various advantages such as safe and enhancement of plant health. This study opens the way for application of safe alternatives to control plant diseases.

## Materials and methods

### Reagents and chemicals

Potato Dextrose Agar (PDA) medium and Difenoconazole fungicide (Daify Core) at extra pure grade, and chemicals were purchased from Sigma-Aldrich and used as received.

### Eggplant seedlings

Three weeks-old eggplant seedlings (*Solanum melongena*) Balady cultivar, obtained from Agricultural Research Center (ARC), Giza, Egypt.

### Source and maintenance of the fungal pathogen

*A. solani* was obtained from Plant Pathology Laboratory, Faculty of Science, Al-Azhar University, Cairo, Egypt. The pathogen was confirmed microscopically, and pathogenicity of isolate confirmed by coukh postulate. To induce sporulation, *A. solani* was cultured in PDA medium for 7 days and incubated at 25 ± 2 °C. Suspensions of conidial were prepared and spore density was counted by a hemocytometer and adjusted to 10^6^ spores/ml. as reported by Boedo et al (2012).

### Isolation and identification of endophytic fungus

Isolation of endophytic fungus was completed by the method of (Aldinary et al. 2021). Healthy *Ocimum basilicum* leaves were obtained from National Research Center, Dokki, Egypt. These leaves were washed and sterilized epiphytically then cultivated on PDA medium supplemented with chloramphenicol (0.2 g/L) and incubated at 27 °C ± 2 for 3 weeks through daily examination, then purified and identified morphologically and molecularly.

### In vitro antifungal activity of endophytic *A. terreus*

Well-diffusion method was applied to study the antifungal activity of ethyl acetate crude extract of endophytic *A. terreus* was inoculated on PD broth medium, then incubated at 28 ± 2 °C for 3–5 days. Fungal inoculum of *A. solani* was spread thoroughly on the sterilized solidified PDA medium. Wells (7 mm) were filled with 100 µl of each fungal extract (10 mg/ml) were put in each well. The culture plates were incubated at 25 °C for 7 days and the zones of inhibition were observed and measured. Moreover, minimum inhibitory concentration (MIC) was carried, where different concentration of each fungal extract (10, 5, 2.5, 1.25, 0.625, 0.3125 and 0.156 mg/ml) was put in wells to detect MIC (Khalil et al. 2021).

### Pot experiment: In vivo study

Applied treatments were added 1 week after infection with *A. solani*, the field trials were conducted at the experimental farm station of Botany and Microbiology Department, Faculty of Science, Al-Azhar University. The pathogenic fungus *A. solani* (10^7^ spores/ml) was introduced into the soil after planting. The inocula suspensions of *A. terreus* was approximately adjusted to 10^9^ CFU/ml culture.

Seedlings were planted in plastic pots (20 × 20 cm) contain 2 kg a mixture of sand and clay soil (1:3) in 5 groups as following: (1) healthy eggplants, (2) infected eggplants with *A. solani*, (3) healthy eggplants treated with *A. terreus*, (4) infected eggplants with *A. solani* and treated with *A. terreus* and (5) infected eggplants with *A. solani* and treated with Daify Core. Disease development and severity were recorded 15 days post inoculation. The plant samples were collected for morphological and biochemical indicators for resistance analysis when the plants were 60 days old.

### Disease symptoms and disease index

Disease symptoms were assessed 60 days after inoculation. While, disease index and protection percent were evaluated according to (Attia et al. 2020). While disease index was evaluated using score consisting of five classes: 0 (no symptoms), 1 (slight-yellowing of lower leaves), 2 (moderate plant-yellowing), 3 (yellow halo around Browne spots) and 4 (concentric rings of raised and depressed dead tissues).

Disease severity (DS) was calculated using the five-grade scale according to the formula: DS % = (1n_1_ + 2n_2_ + 3n_3_ + 4n_4_)100/4n_t_. Where n_1_–n_4_ indicate the number of plants in the indicated classes, and nt indicates the total number of tested plants.

Percent protection by PGPR strains was calculated using the following formula:

$${\text{Protection}}\% \, = \,{\text{A}}{-}{\text{B}}/{\text{A}}\, \times \,{1}00\%$$

where, A = PDI in non-inoculated control plants and B = PDI in treated plants.

### Biochemical indicators for eggplant resistance

Determination of photosynthetic pigments carried out according to Vernon and Seely (2014; Badawy et al. 2021a). Briefly, fresh 0.5 g leaf tissue was crushed in acetone (80%) using a pestle and mortar to estimate the pigment content. After centrifuging the filtrate for 5 min at 10,000 × g, the absorbance of the filtrate was measured at 470, 652, and 665 nm to estimate chlorophyll a, chlorophyll b and carotenoid content.

A described method of Umbreit et al. (1964) was applied for determination of carbohydrate contents in the dried tissues of eggplants. Briefly, the dried shoots (0.5 g) diluted with 5 ml of 30% trichloroacetic acid (TCA) and 2.5 ml of 2% phenol and filtered through filter paper, then 1 ml of the filtrate was treated with 2 ml of anthrone reagent (2 g anthrone/L of 95% H_2_SO_4_). 620 nm was used to determine the produced blue green color.

Contents of soluble proteins were calculated according to Lowry et al*.,* (1951). Briefly, 1 mL of plant extract was combined with 5 mL of alkaline reagent (50 ml of 2% Na_2_CO_3_ prepared in 0.1 N NaOH and 1 ml of 0.5% CuSO_4_ prepared in 1% potassium sodium tartrate) and 0.5 ml of Folin’s reagent (diluted by 1:3 v/v). After 30 min, a color change could be seen at a wavelength of 750 nm.

While, the well-established method of Dai et al. (1993) was used to assay the total phenolics. One gram of plant tissue was extracted in 5–10 ml of ethanol (80%) for at least 24 h. After filtration, the residue was re-extracted twice times with the same solvent. All extracts were completed to 50 ml with ethanol (80%). The extract (0.5 ml) was mixed well with 0.5 ml of Folin’s reagent followed by shaking for 3 min. Saturated Na_2_CO_3_ solution (1 ml) then distilled water (3 ml) were added and homogenized well. After 1 hour, the developed blue color was measured using a spectrophotometer at 725 nm.

The described method of Bates et al., (1973) was used to free proline content determination. In such a method, 0.5 g of dried plant material was homogenized in 10 ml of sulfosalicylic acid (3%). After filtration, 2 ml of filtrate reacted with 2 ml acid ninhydrin (1.25 g of ninhydrin in 30 ml of glacial acetic acid and 20 ml of 6 M phosphoric acid, with agitation, until dissolved) and 2 ml of glacial acetic acid. This reaction was placed in a boiling water bath for 1 hour, then placed in an ice bath. Finally, the reaction mixture extracted with 4 ml of toluene. The chromophore containing toluene was aspirated from the aqueous phase and read spectrophotometrically at 520 nm.

Malondialdehyde contents determined according to Abd Alhakim et al.(2022). The MDA content was determined according to its molar coefficient of absorbance of 155 mmol L^−1^ cm^−1^ and expressed as nmolg^−1^ FW. Superoxide dismutase and catalase activities were determined by methods described by Bergmeyer (1974).

Adopted method of Srivastava et al*.,* was used to determine peroxidase activity (Srivastava 1987). Eventually, the activity of polyphenol oxidase was measured by the adopted method from Matta (1969).

### Statistical analyses

One-way variance analysis (ANOVA) was applied to the resulting data. Least significant difference (LSD test) using CoStat (CoHort, Monterey, CA, USA) was used to demonstrate statistically relevant differences between treatments at p < 0.05. Results shown as mean ± standard errors (n = 3) (Snedecor and Cochran 1982).

## Results

### Identification of the endophytic fungus

**Fig. 1:**
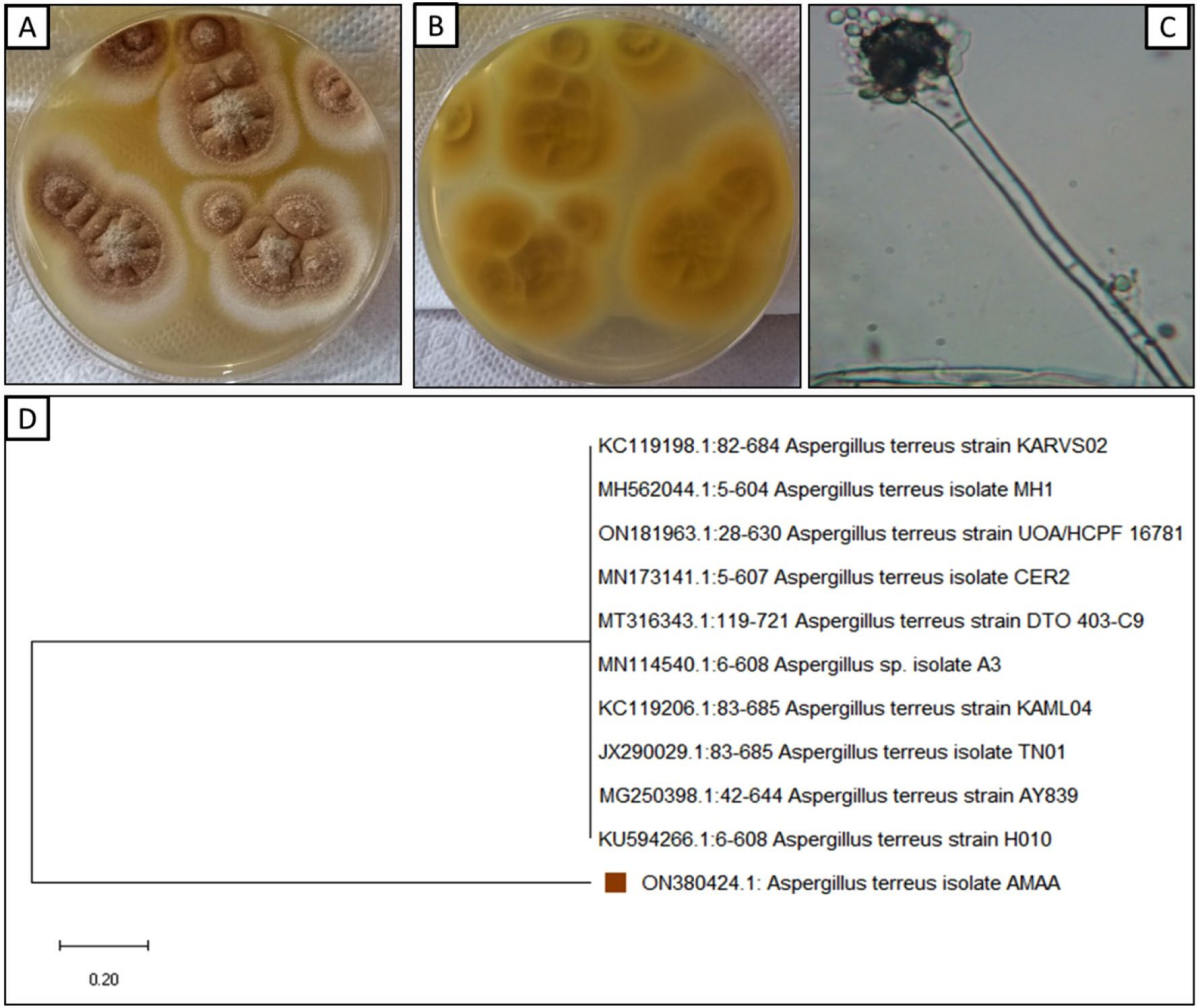
**A**- Colony of *A. terreus* on PDA grown at 28 °C for 4 days showing the culture characteristics; **B**- Light microscope showing stipe, conidia, strigmata and conidial head of *A. fumigatus* (400X); **C**- Phylogenetic tree of *A. terreus* with accession number ON380424

Macroscopically identification illustrated low growth frequency with finely granular conidial creation at 27 ± 2 °C on PDA medium 20–35 mm diameter after 7 days. Surface buff with reverse yellow to orange pigments dark in center due to the presence of Cleistothecia surrounded by yellow Hull cells (Fig. 1A). Microscopically, mycelium is septated, conidiophores ranging 70 to 300 μm with smooth-walled, ending with globose vesicles. Pyriform vesicles, 8–12 mm containing hyaline small conidia (2–2.5 μm), globose, and smooth (Fig. 1B). Molecular identification using ITS gene confirmed that this endophytic fungus is resemble to *Aspergillus terreus* with similarity 99% respectively. The sequence was recorded in GenBank with accession number ON380424. (Fig. 1D).

### Antifungal activity

**Fig. 2:**
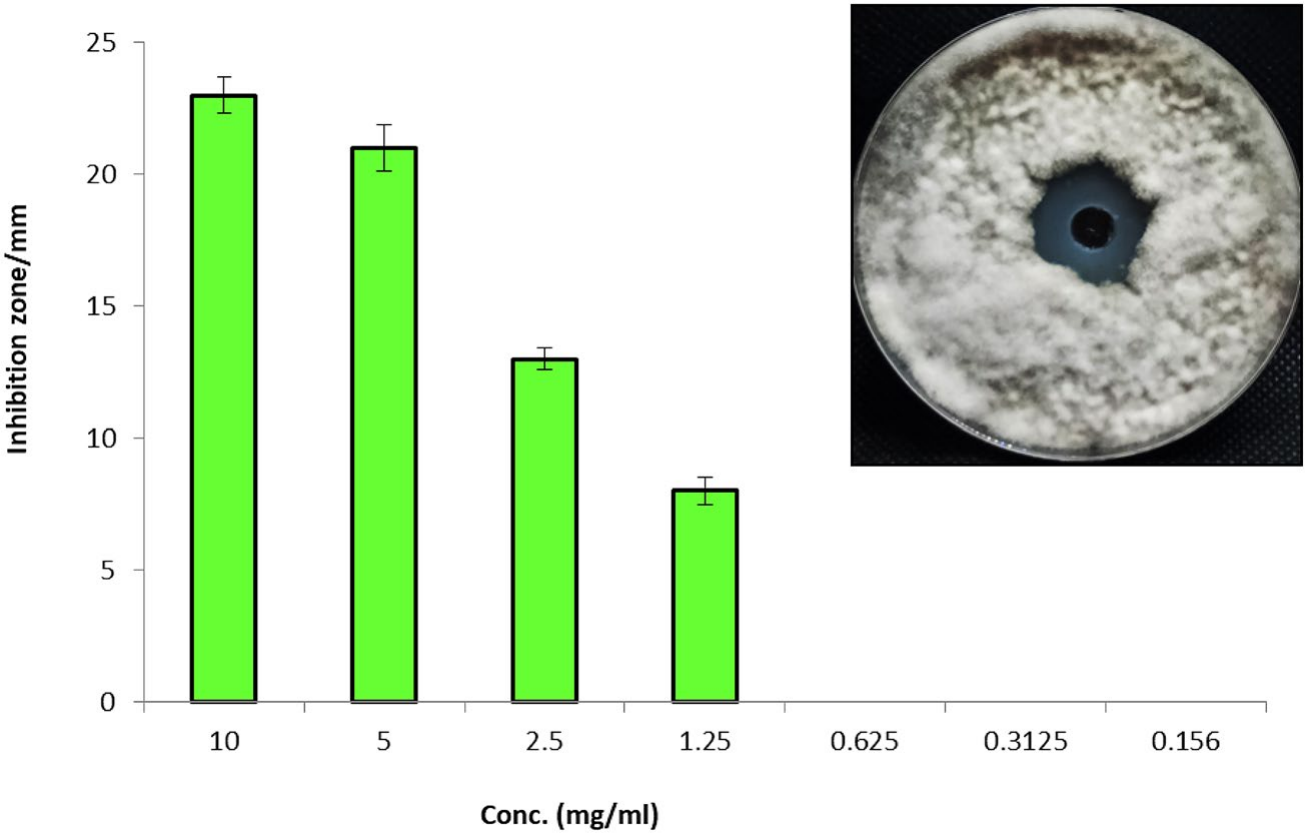
Antifungal activity of *A. terreus* against *A. solani* invitro efficacy of *A. terreus* on *A. solani* early blight disease of *S. melongena* under pots conditions

Antifungal activity of ethyl acetate crude extract of endophytic *A. terreus* was evaluated against *A. solani* using agar well diffusion method. Results illustrated that, endophytic *A. terreus* extract exhibited potential antifungal activity toward *A. solani* where inhibition zone of the extract at concentration 10 mg/ml was 23 mm. Additionally, different concentrations of the extract were evaluated as antifungals against A. solani, where result showed that inhibition zones were 21, 13, 8 mm at 5, 2.5 and 1.25 µg/ml respectively (Fig. 2). On other hands, concentrations below 1.25 µg/ml did not give any inhibition on the growth of *A. solani*. Consequently, the concentration 1.25 µg/ml is the MIC of endophytic *A. terreus* extract.

Results presented in Table 1 indicated that *A. solani* caused the emergence of severe symptoms on the *S. melongena* plant 87.5% compared with healthy control. The results also showed that chemical fungicide (Daify Core) and* A. terreus* highly caused recovery of early blight with highly protection by 76.19% and 66.67%.

**Table 1:** Effect of *A. terreus* on *A. solani* early blight disease of *S. melongena*

Treatment	Disease symptoms classes					DI (disease index) (%)	Protection (%)
	0	1	2	3	4		
Control healthy	6	0	0	0	0	0	–
Control infected	0	0	0	3	3	87.5	0
Infected treated with *A. terreus*	1	3	2	0	0	29.16	66.67
Infected treated with **(**daify core)	3	1	2	0	0	20.83	76.19

### Vegetative growth

**Fig. 3:**
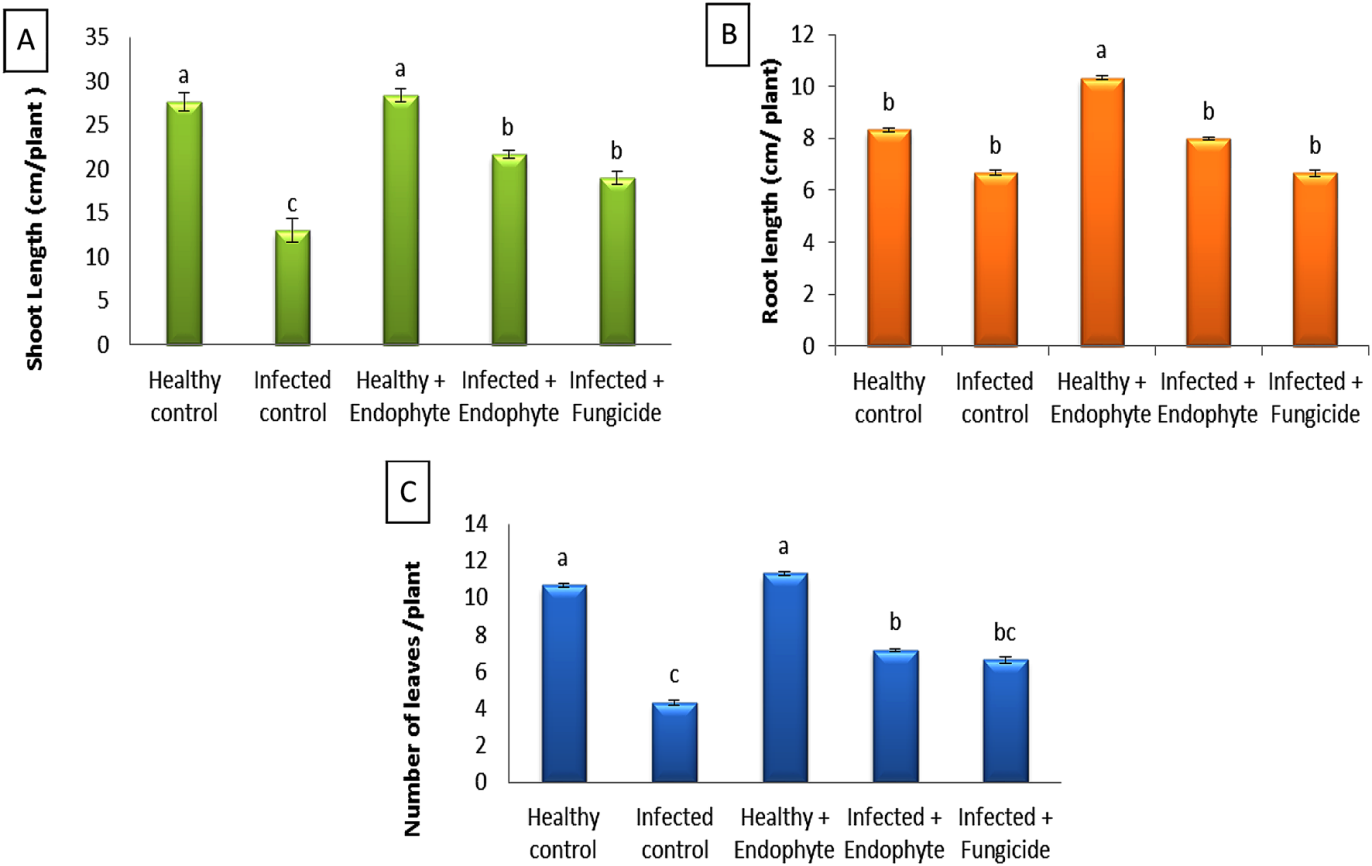
Effect of *A*. *terreus* on **A** shoot length, **B** root length and **C** number of leaves of *S. melongena*. Each bar represents mean ± standard error

Results in Fig. 3 indicated that, *A. solani* caused highly significant decreasing of eggplant growth including shoot length, root length and number of leaves by (53.00%, 20.04% and 59.38). On the other hand, application of *A. Terreus* resulted to enhancement of both healthy and infected plants compared to control.

### Photosynthetic pigments

**Fig. 4:**
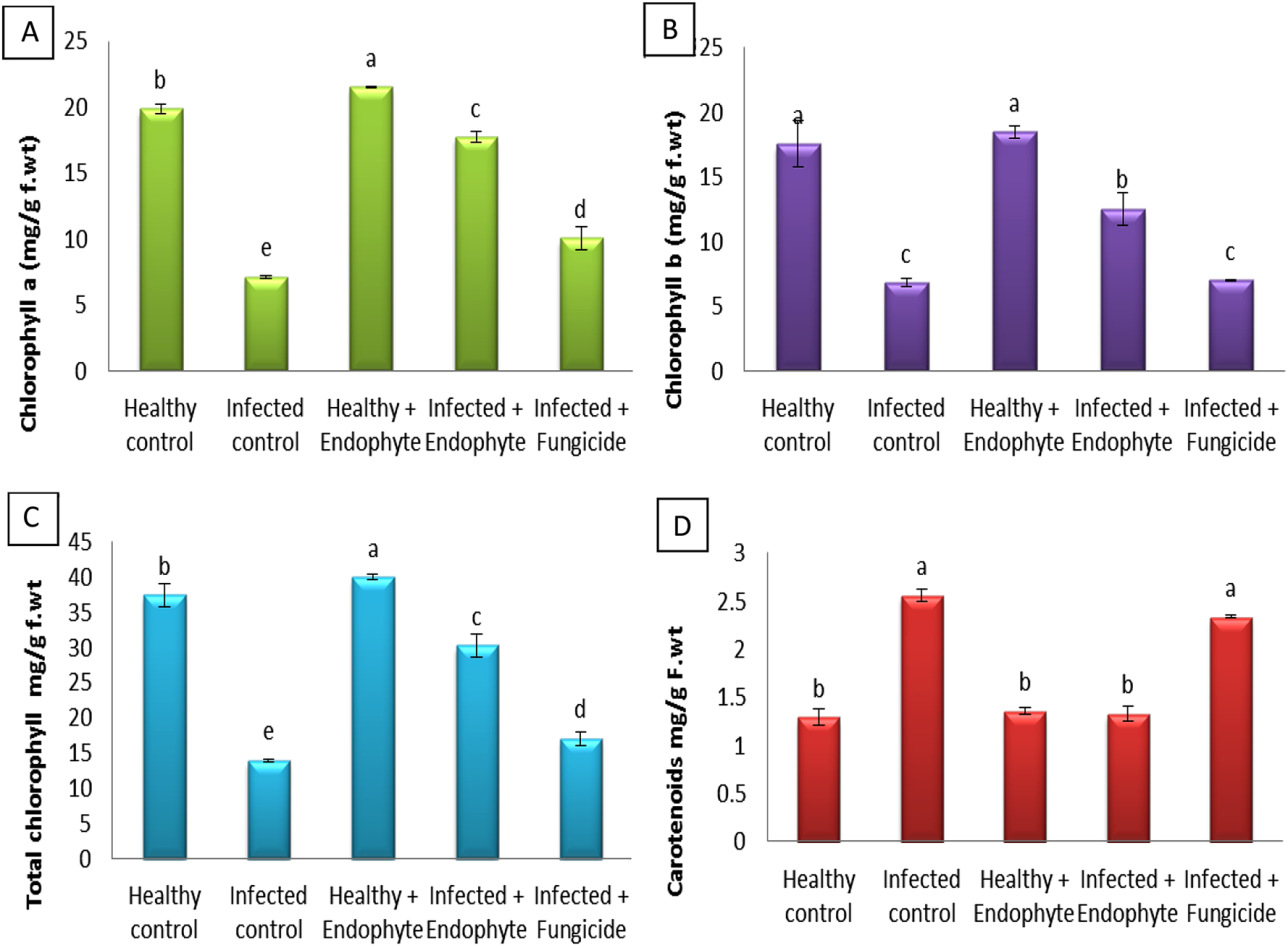
Effect of *A*. *terreus* on **A** chlorophyll *a*, **B** chlorophyll *b*, **C** total chlorophylls and **D** carotenoids of *S. melongena*

The observed results in Fig. 4 showed that the infection of eggplant seedlings with *Alternaria* caused a severe deficiency of the photosynthetic pigments; chlorophyll *a* and *b* by 63.93% and 60.88%, respectively, and a significant increase in the level of carotenoids compared to healthy plants. It is interesting that the treatment of eggplant seedlings, whether healthy or infected with *A. terreus*, showed a clear and noticeable improvements in photosynthetic pigments (chlorophyll *a* and *b*). Results in Fig. 4 indicated that the *A. terreus* treatment was better than the fungicide, as the plants treated with *A. terreus* recorded an increase in chlorophyll *a* and *b* (147.95% and 83.279%) compared to the fungicide (40.79% and 2.33%). But the application of fungicide to the infected plants led to a significant increase in the content of carotenoids compared to infected plants treated with *A. terreus*.

### Metabolic indicators

It was observed from Table 2 that carbohydrate contents in eggplants were significantly reduced by about 29.94% as a result of the infection with the pathogenic fungus *A. solani*. On the other side, the endophyte *A. terreus* application significantly increased the contents of carbohydrates in healthy eggplants by 13.21%. Regarding the infected eggplants, application of the fungus endophyte representing in *A. terreus* and the chemical fungicide minimized the harmful effects of the pathogenic *A. solani*. The endophyte *A. terreus* was more effective by 27.13% than the fungicide 19.01%.

**Table 2:** Effect of *A*. *terreus* on carbohydrates, proteins, phenols and proline (mg g^−1^ DW) of *S. melongena*. Each value represents mean ± standard error

Treatments	Total soluble carbohydrate	Total soluble protein	Phenol	Free proline
Control healthy	64.71 ± 1.55^b^	1.9 ± 0.01^ab^	1.58 ± 0.02^d^	6.51 ± 0.06^d^
Control infected	45.33 ± 1.38^d^	1.7 ± 0.03^d^	2.8 ± 0.03^a^	8.50 ± 0.04^a^
Healthy treated with *A. terreus*	73.26 ± 2^a^	2 ± 0.02^a^	1.79 ± 0.02^c^	7.07 ± 0.05^c^
Infected treated with *A. terreus*	57.63 ± 2.52^c^	1.8 ± 0.02^bc^	2.11 ± 0.1^b^	7.49 ± 0.26^b^
Infected treated with fungicide (daify core)	53.95 ± 2.56^c^	1.8 ± 0.02^c^	2.14 ± 0.04^b^	7.75 ± 0.05^b^

Impact of the pathogenic fungus *A. solani* on the contents of protein in eggplants and their management by endophyte and fungicide were clarified in Table 2. It was clearly revealed that protein contents in eggplants that were infected with *A. solani* were significantly decreased by about 8.95% less than uninfected plants (healthy control). Individual application with *A. terreus* obviously enhanced the contents of protein in healthy eggplants. In case of the infected eggplants, application of *A. terreus* and chemical fungicide significantly enhanced protein contents about 6.36% and 5.88%.

Phenolic compounds of eggplants were affected due to the infection with *A. solani* and the application of endophyte *A. terreus* and chemical fungicide (Table 2). Pathogenic fungus *A. solani* significantly augmented the contents of phenols in eggplants by about 77.22% relative to uninfected plants. Additionally, endophyte *A. terreus* significantly enhanced phenolic compound contents by about 13.29% relative to healthy control plants. In case of the infected eggplants, phenols contents were significantly increased relative to healthy control plants in response to the application with endophyte and fungicide by 33.54% and 35.44%, respectively, but phenolics significantly decreased relative to infected control plants due to the application with endophyte and fungicide by 24.64% and 23.57%, respectively.

Also, free proline contents in eggplants were changed in response to *A. solani* infection and the application of endophyte *A. terreus* and chemical fungicide as shown in Table 2. Infection with the pathogenic fungus *A. solani* significantly boosted proline contents in eggplants by 30.57% in comparison with uninfected plants. Individual treatment with the endophytic fungus *A. terreus* significantly promoted proline contents by 8.60% when compared with healthy control plants. Regarding to the infected eggplants, proline contents were significantly enhanced in response to the application with endophyte and fungicide by 15.05% and 19.05%, respectively, comparing with healthy control eggplants but significantly decreased due to the application with endophyte and fungicide by 11.88% and 8.82%, respectively, when compared with the infected control eggplants.

### Malondialdehyde (MDA)

**Fig. 5:**
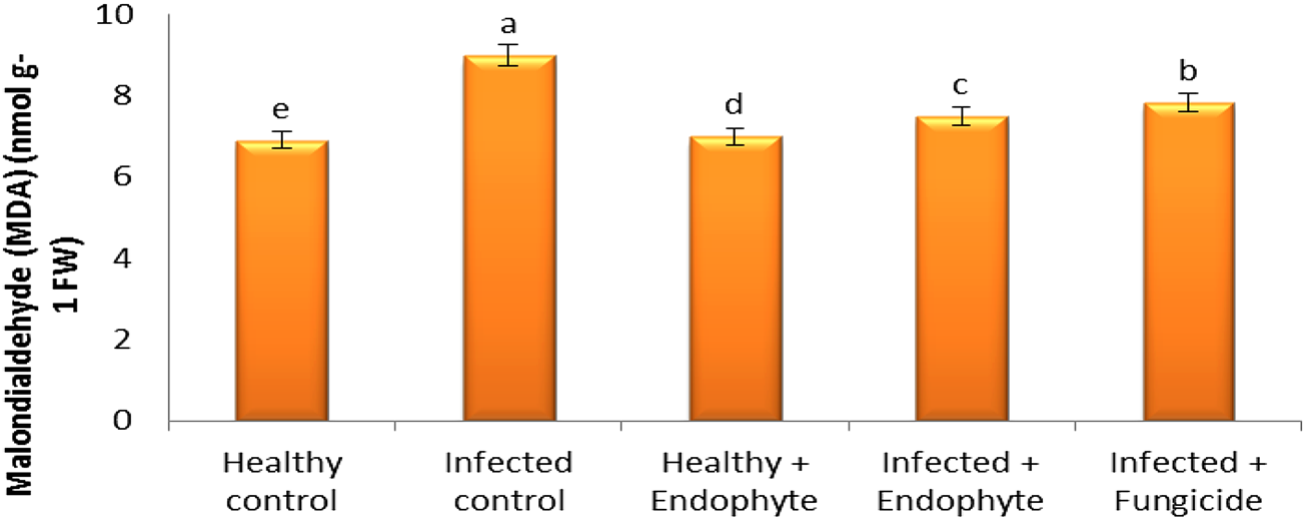
Effect of *A*. *terreus* on Malondialdehyde of *S. melongena*

Infection with *Alternaria* led to an increase in MDA accumulation compared to healthy plants (Fig. 5). A clear decrease in the level of MDA as a result of the use of *A. terreus* and fungicide on infected plants where the treatment with *A. terreus* was better than the fungicide in reducing the level of MDA by 16.6% and 12.82%.

### Antioxidant enzymes activity

**Fig. 6:**
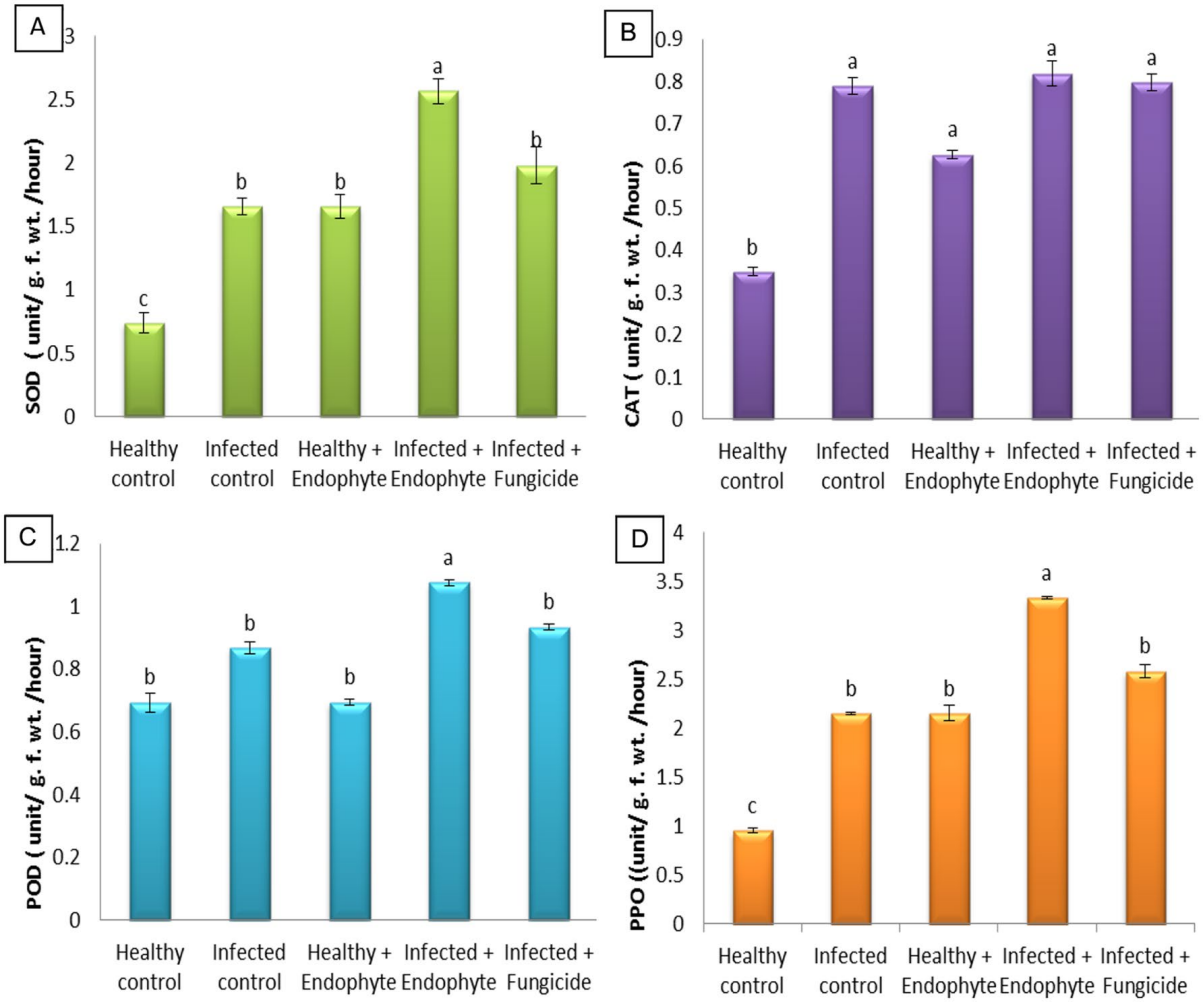
Effect of *A*. *terreus* on **A** superoxide dismutase, **B** catalase, **C** peroxidase and **D** polyphenol oxidase of *S. melongena*. Each bar represents mean ± standard error

The activities of antioxidant enzymes (SOD, CAT, POD and PPO) in infected eggplant seedlings with *A. solani* and healthy were recorded in Fig. 6. To obtain a clearer indication of the occurrence of resistance, the activities of the antioxidant enzymes were measured in seedlings of eggplant treated with *A. terreus* and the fungicide. Results in Fig. 6 indicated that the addition of *A. terreus* to the healthy plants led to a significant increase in the activities of SOD, CAT, POD and PPO compared to the healthy plants. Interestingly, the addition of *A. terreus* to healthy plants stimulated the activation of antioxidant enzymes more than the fungicide**.**

### Isozymes

**Fig. 7:**
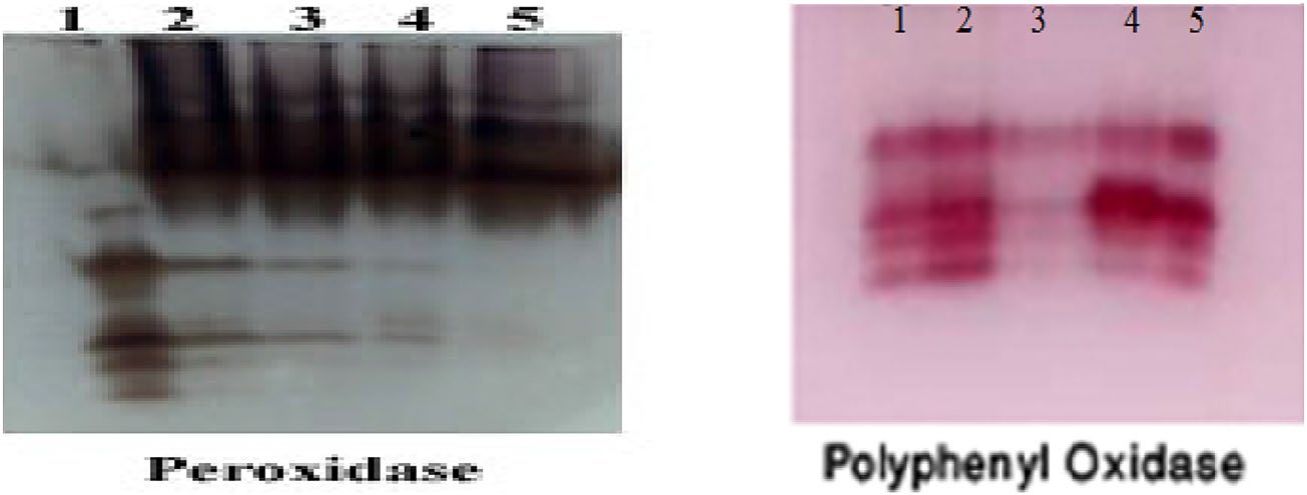
Effect of *A*. *terreus* on antioxidant isozymes activity of *S. melongena*

By assessing isozymes of antioxidants, the results showed that infection with Alternaria fungus induced highly express peroxidase isozymes (Table 3 and Fig. 7). Application of *A. terreus* to both healthy and infected plants showed recorded the same bands, 5 bands, 2 of them are very strong at RF (0.210 and 0.343) and 3 moderated bands at Rf (0.134,0.583 and 0.782). Also, addition of chemical Fungicide to infected plants showed 5 bands, 2 of them are very strong at RF (0.210 and 0.343) and 2 moderated bands at Rf (0.134 and 0782) and 1 strong band at RF (0.446). Uninfected plants and treated with (*A. terreus*) expressed the lowest POD expression that they produced 2 faint bands at Rf (0.484 and 0.607,) and 1 moderate band at (0.806). Polyphenol oxidase isozyme antioxidants showed maximum expression at untreated infected plants with *Alternaria* as compared to other treatments (Table 4 and Fig. 7). Enzymatic antioxidant (PPO) highly posed expression in response to *A. terreus*, and chemical fungicide. In other words, chemical fungicide showed maximum expression as compared *A. terreus*. Uninfected plants and treated with *A. terreus* expressed the lowest PPO expression that they produced 3 faint bands.

**Table 3:** Effect of *A*. *terreus* on peroxidase isozyme of *S. melongena*

RF	Lane1	Lane2	Lane3	Lane4	Lane5
0.134	−	+	+	+	+
0.210	−	+ + +	+ + +	+ + +	+ + +
0.343	+	+ + +	+ + +	+ + +	+ + +
0.446	+	+	−	−	+ +
0.583	+	+	+	+	−
0.782	+ +	+	+	+	+
0.850	+	−	−	−	−
0.935	+	−	−	−	−

**Table 4:** Effect of *A*. *terreus* on polyphenol oxidase isozyme of *S. melongena*

RF	Lane1	Lane2	Lane3	Lane4	Lane5
0.3	−	+ +	+	+	+ +
0.4	+	+ +	+	+ +	+ + +
0.5	+	+ + +	+	+	+
0.6	+	+ +	−	+	+

## Discussion

Fungal endophytes are considered one of important reservoirs of bioactive compounds which have different biological activities such as antimicrobial, antioxidant, anticancer, antiviral and antimalarial activities (Sharaf et al. 2022; Khalil et al. 2021). These activities are attributed to different effective secondary metabolites as alkaloids, phenols, steroids, terpenoids, saponins, glycosides, tannins and flavonoids (Ancheeva et al. 2020; Kouipou Toghueo and Boyom 2019). In this study, an endophytic fungus was isolated from *Ocimum basilicum* leaves and was identified as *A. terreus* morphologically and genetically, where it was recoded with accession number ON380424. A previous study isolated *A. terreus* from different plants, which can be used as antibacterial and antifungal agents (Hashem et al. 2022). Endophytic *A. terreus* fungus was used previously for bio-control of sesame wilt disease (Hegazy et al. 2019), Pythium-induced damping-off of cucumber (Halo et al. 2018) and okra-rot-causing *Cochliobolus spicifer*-CSN-20 (Abdel-Motaal et al. 2022). In our study, endophytic *A. terreus* was used for bio-controlling of *A. solani* which causes early blight disease. Results revealed that the extract of endophytic *A. terreus* has promising antifungal activity against *A. solani.*

Fungal infection causes a detrimental effect on vegetative growth characteristics, which negatively reflect on all physiological processes. Our results in the current experiment showed that the fungus *Alternaria* caused a severe decrease in vegetative growth characteristics. The results of the current study agree with (Alhaithloul et al. 2019; Orcutt and Nilsen 2000) they concluded that infection with *Alternaria* causes a sharp decrease in the vegetative growth characteristics of many plants. This sharp decrease in growth characteristics because of infection with *Alternaria* can be explained by the severe disturbance in growth hormones and the oxidative explosions that occur inside the cells as a result of the infection (Attia et al. 2017; Bos 1978). One of the most important manifestations of disease resistance is the improvement of vegetative characteristics such as stem length, root length, and number of leaves. Our results indicated that the treatment with Daify core or *A. terreus* improved eggplant resistance represented by 76.19% and 66.67% protection against *Alternaria* infection. Also, our results showed that the endophytic fungus *A. terreus* was more effective in improving vegetative growth characteristics, whether infected or healthy eggplants. This improvement can be explained by the fact that *A. terreus* contains stimulating compounds for plant growth, in addition to its anti-fungal ability that induce the growth of plants under unfavorable conditions (Ismail et al. 2020; Mousa 2016).

Photosynthesis is one of the most important vital activities within the plant. The results of the current recorded a severe deficiency of photosynthesis pigments because of infection with the fungus *Alternaria*; this is due to the failure of the plant to capture light and the breakdown of chlorophyll pigments. This means that the plant will fail in capturing sunlight and thus photosynthesis will be decreased or inhibited (Sharma et al. 2012; Gámez-Arcas et al. 2021). It is interesting that the treatment of infected or healthy plants with *A. terreus* led to a clear and noticeable improvement in chlorophyll pigments. It is interesting that the treatment of infected or healthy plants with *A. terreus* led to a clear and noticeable improvement in chlorophyll pigments, which reinforces the idea of using endophytic fungus* A. terreus* to treat the damage caused by fungal infection, as well as plant growth stimulants. The results of this study are in agreement with (Aldinary et al. 2021), which indicated that the use of fungal endophytes increases and improves the efficiency of the photosynthesis process, due to many changes in the chloroplasts and the contents of carotene and chlorophyll.

It has been demonstrated that total sugar contents were reduced in different varieties of mustard plants in response to Alternaria blight infection stress (Mallick et al. 2015). On the other side, and in parallel with our results, application of the endophytic fungus *Aspergillus ochraceus* led to significant enhancements in sugar content in barely plants (Badawy et al. 2021b). A recent study on early blight disease documented that *Aspergillus flavus* treatment caused significant increases in carbohydrate content in tomato plants when compared with untreated plants (Abdel-Motaal et al. 2020). On the same line, levels of soluble carbohydrates were significantly enhanced in powdery mildew-infected berry leaves in response to fungicide (Trinolfungicide) treatment (Fayez et al. 2016). It was suggested that accumulation of carbohydrates in plant tissues play a protective role under biotic and abiotic stress condition as well as cell wall supporting and antioxidant production (Abdel Latef et al. 2021; Keunen et al. 2013).

A similar study documented a reduction in the protein content in the Alternaria-infected eggplants when compared with the uninfected plants (Alhaithloul et al. 2019). In agreement with our findings, utilization of the endophytic fungus *A. ochraceus* as a biotic inducer was found to increase the amount of soluble protein in barely plants (Alhaithloul et al. 2019). A study of Abdel-Motaal et al. (2020) on early blight disease in tomato plants displayed significant improvements in protein contents as a result of *Aspergillus flavus* treatment in comparison with untreated plants. Also, application of chemical fungicides markedly increased protein contents in the infected berry leaves (Fayez et al. 2016).

Phenolic compounds play a protective role in plants exposed to either abiotic or biotic stress (Adhikari et al. 2017; Hussein et al. 2022; Matta 1969; Osman et al. 2021). Accumulation of these compounds in infected plants by fungal pathogens has been recorded in several studies (El-Khallal 2007; Ramamoorthy et al. 2002). It was recently documented that the contents of total phenolic compounds were significantly increased in the infected tomato plants by *A. cerealis* in individual treatments (Mahmoud et al. 2021). Also, *A. brassicae* infection caused significant increases in phenol content in some mustard varieties (Mallick et al. 2015). Regarding the interaction, a similar result indicated that phenolic compounds were accumulated in tomato plants that were infected with the pathogenic fungus *A. cerealis* and treated with *Trichoderma harzianum* compared with control plants (Mahmoud et al. 2021). Moreover, phenols content significantly promoted in powdery mildew-infected berry leaves when treated with chemical fungicides (Fayez et al. 2016). These results suggest that each enhancement or accumulation in phenol contents induces systemic resistance in the host to face the stress.

Under such as proline that act as osmo-regulator environmental stress either biotic or abiotic, plants accumulate osmolytes to scavenge reactive oxygen species as well as to cope with these different stresses (Li et al. 2017; Mallick et al. 2015). Data in the present study revealed that proline content in *A. solani*-infected eggplants were increased. Similarly, it has been reported that free proline contents were significantly accumulated in tomato tissues which infected by *A. solani*, the causative agent of early blight disease (Kumari et al. 2017). Also, levels of free proline were increased in some mustard varieties under Alternaria blight infection stress (Mallick et al. 2015). Regarding application of *A. terreus* endophyte, previous studies have documented that plant growth-promoting fungi induce systemic protection against phytopathogens (Fontana et al. 2021; Kim et al. 2007). In addition, stressed plants that were treated with fungal endophytes (such as *Piriformospora indica* and *Aspergillus ochraceus*) had low levels of proline in comparison with non-treated plants (Abdelaziz et al. 2019; Badawy et al. 2021b). Our findings are in accordance with the results of Fayez et al. (2016); the author demonstrated that fungicide (Trinolfungicide) treatment increased proline levels in the infected berry leaves in comparison with healthy control plants while decreased these levels in the infected berry leaves comparing with the infected control plants. These findings suggest that application of fungal endophyte ameliorate the probable stress that located on plants.

The biotic and a biotic stresses caused intracellular oxidative stress, which led to severe disturbance in the plant cell and an increase in the content of MDA (Dallagnol et al. 2011; Badawy et al. 2021b). Infection with *Alternaria* fungus led to an increase in MDA accumulation compared to healthy plants. On the other hand, the addition of fungal endophyte to the affected plants showed a significant decrease in the content of MDA, and this can be explained by ability fungal endophyte to induce systemic resistance by increasing the antioxidants responsible for defense to reduce oxidative stress within cells (Badawy et al. 2021b).

Several antioxidant defense enzymes (peroxidase, superoxide dismutase, catalase, and polyphenol oxidase) have been associated with infection (Sofy et al. 2020; Jiang et al. 2019). The results of this study dealt with the estimation of the activity of antioxidant defense enzymes and indicated that infection with *Alternaria* fungus caused a significant increase in enzymes (SOD, CAT, POD and PPO) compared to healthy plants. On the other hand, the use of fungal endophyte or fungicide on infected plants induced the plant to increase enzymatic activity as a means of defense. Our results showed that antioxidant enzymes activity increased significantly in plants exposed to *Alternaria* infection. The plant showed different approaches to cope with infection as they increased the activity of certain antioxidant enzymes to keep ROS at the lower level in the cell. Antioxidant enzymes as POD help in the conversion of H_2_O_2_ to H_2_O (Gill and Tuteja 2010).

Antioxidant isozymes play an important role in defending against different stresses and risks to which plants are exposed as they control the metabolism process and protect cells from oxidative stress (Attia et al. 2016). By assessing isozymes of antioxidants, the results showed that infection with *Alternaria* fungus induced highly expressed peroxidase isozymes. Application of *A. terreus* to both healthy and infected plants recorded the highest expression of (POD and PPO) Isozymes. Inducing the plant to produce these enzymes is one of the most important means of defense against stress (Abdelaziz et al. 2022c).

## Conclusion

From the present study, Endophytic *A. terreus* ON380424 was isolated from *O. basilicum* healthy leaves, and it can be used as a hopeful and safe alternative antifungal agent against *Alternaria solani* in vitro and in vivo. However, the promising results obtained from our study reveals that, Endophytic *A. terreus* ON380424 increasing the induction of the systemic resistance of the eggplant. *A. terreus* isolate reduced disease severity by 29.16.0% and recorded highly protection by 66.67% when compared to non-treated plants. The infected plants treated with *A. terreus*, showed the most potent effect in terms of the length of shoots and roots and the number of leaflets per plant. Additionally, eggplant which were treated with endophytic *A. terreus* ON380424 showed a significant increase in the content of chlorophyll a and b and carotenoids, the total phenols, free proline, total soluble proteins, POD and PPO compared to the non-treated infected plant. These results are interesting, as the percentage of protection from disease converges as a result of the use of endophytic *A. terreus* ON380424 (66.67%) compared to the fungicide **(**76.19%). On the other hand, it is beneficial and enhances plant immunity and is safe for plant and human health. Accordingly, endophytic *A. terreus* ON380424 is a promising isolate for potential applications in food processing and packaging, agricultural application and as a smart biological control against *A. solani* which causing early blight disease of eggplant.

## Data Availability

All data and materials viable.
